# The Hazards of Probiotics on Gut-Derived *Pseudomonas aeruginosa* Sepsis in Mice Undergoing Chemotherapy

**DOI:** 10.3390/biomedicines12020253

**Published:** 2024-01-23

**Authors:** Fu-Chen Huang, Shun-Chen Huang

**Affiliations:** 1 Department of Pediatrics, Kaohsiung Chang Gung Memorial Hospital and Chang Gung University College of Medicine, Kaohsiung 833, Taiwan; 2 Department of Anatomic Pathology, Kaohsiung Chang Gung Memorial Hospital and Chang Gung University College of Medicine, Kaohsiung 833, Taiwan; shuang@cgmh.org.tw

**Keywords:** probiotics, *Pseudomonas aeruginosa*, sepsis, chemotherapy, mice model

## Abstract

*Pseudomonas aeruginosa* (*P. aeruginosa*) is a leading cause of nosocomial infections associated with a high mortality rate and represents a serious threat to human health and the increasing frequency of antimicrobial resistance. Cancer patients are more vulnerable to invasive infection due to ulcerative lesions in mucosal surfaces and immune suppression secondary to chemotherapy. In our in vitro study, we observed that probiotics have the potential to yield beneficial effects on intestinal epithelial cells infected with *P. aeruginosa*. Additionally, probiotics were found to confer advantageous effects on the innate immunity of mice suffering from *Salmonella*-induced colitis. As a result, we sought to investigate the impact of probiotics on gut-derived *P. aeruginosa* sepsis induced by chemotherapy. Following chemotherapy, gut-derived *P. aeruginosa* sepsis was induced in female C57BL/6 mice aged 6–8 weeks, which were raised under specific-pathogen-free (SPF) conditions in an animal center. Prior to the induction of the sepsis model, the mice were administered 1 × 10^8^ colony-forming units (CFU) of the probiotics, namely *Lactobacillus rhamnosus GG* (LGG) and *Bifidobacterium longum* (BL) via oral gavage. We observed that LGG or BL amplified the inflammatory mRNA expression in mice undergoing chemotherapy and suffering from gut-derived *P. aeruginosa* sepsis. This led to a heightened severity of colitis, as indicated by histological examination. Meanwhile, there was a notable decrease in the expression of antimicrobial peptide mRNA along with reduced levels of zonulin and claudin-2 protein staining within mucosal tissue. These alterations facilitated the translocation of bacteria to the liver, spleen, and bloodstream. To our astonishment, the introduction of probiotics exacerbated gut-derived *P. aeruginosa* sepsis in mice undergoing chemotherapy. Conclusively, we must be prudent when using probiotics in mice receiving chemotherapy complicated with gut-derived *P. aeruginosa* sepsis.

## 1. Introduction

*Pseudomonas aeruginosa* (*P. aeruginosa*) is a leading cause of nosocomial infections in intensive care units (ICUs), such as ventilator-associated pneumonia, bloodstream infections, urinary tract infections, or superinfections of burn wounds associated with mortality rates of more than 30% [1,2], leading to lengthened hospital stays, and it is also a leading cause of the development of multidrug-resistant *P. aeruginosa*, making this pathogen one of great concern. Because of the increasing frequency of antimicrobial resistance (AMR) in *P. aeruginosa*, the World Health Organization has rated multidrug-resistant (MDR) *P. aeruginosa* as serious threat for human health and emphasized the urgent need for new treatment options. The increased failure frequency of antibiotic treatments raises breakthrough strategies surpassing classical antibiotic usage are therefore needed to overcome AMR. So-called host-directed therapies (HDTs) [3], enhancing protective immune signatures, reducing exacerbated inflammation, or balancing immune reactivity at the infection site, represent one of the major alternative approaches against AMR. Indeed, antibiotic usage is a risk factor for *P. aeruginosa* resistance, and it would thus be highly important to develop a useful strategy to prevent and limit the use of antibiotics. A meta-analysis provided evidence that probiotic use has the potential to decrease antibiotic utilization in children [4]. Moreover, effective immunotherapy may be a useful alternative therapy administered either alone or in combination with antibiotic chemotherapy.

Cancer patients are more vulnerable to invasive infection due to ulcerative lesions in mucosal surfaces and immune suppression secondary to chemotherapy [5]. Studies conducted in oncology–hematology units found a high intestinal carriage of *P. aeruginosa* between 11.7 and 37% [6]. The translocation of endogenous intestinal *P. aeruginosa* extraluminally is an important cause of systemic infections, especially in neutropenic patients with hematological malignancies [7]. *P. aeruginosa* bacteraemia is well known for its rapid progression into fatal sepsis and is associated with a high rate of morbidity and mortality in immunocompromised hosts, such as those suffering from chemotherapy-induced neutropenia [8]. Infections remain among the most-encountered serious complications of chemotherapy treatment among pediatric patients with malignancies, accounting for morbidity and mortality [9,10]. Sometimes infectious complications lead to an early death before cancer remission can be achieved, in spite of the use of prophylactic antibiotics [10].

After administering an oral challenge using a clinical *P. aeruginosa* isolate, stable colonization of the intestines by *P. aeruginosa* was achieved several days post infection. Strikingly, cytokine responses upon intestinal *P. aeruginosa* colonization were not restricted to the intestinal tract but could also be observed systemically [11]. This implies that intestinal mucosa, first coming into contact with the pathogen, have a central part in the innate immune response against *P. aeruginosa* infection. The disruption of mucosal barrier and colonization resistance may be partly responsible for the bacterial translocation during chemotherapy [12]. Because a disrupted intestinal microbiota might be associated with a higher susceptibility to sepsis and a poor outcome, microbiota-targeted interventions during critical illness should be increasingly investigated.

The findings from a meta-analysis, which included 13 randomized controlled trials (RCTs) encompassing 1024 patients, indicated that the utilization of probiotics either before or during chemotherapy proved to be a successful preventive measure against chemotherapy-induced diarrhea (CID) in cancer patients [13]. Additionally, none of the studies included in the analysis reported any adverse reactions associated with the administration of probiotics. In a review paper from 2020, it was concluded that probiotics demonstrate utility in mitigating the risk of various types of cancer and aiding in the management of existing chemotherapy-related side effects. However, the majority of the positive outcomes observed are predominantly confined to experimental settings rather than extensive clinical evidence [14]. Moreover, a Cochrane review referenced as [15] did not discover any substantial evidence supporting the efficacy of treatment. The available evidence remains inconclusive regarding the prevention of chemotherapy-induced diarrhea, although no adverse effects were noted. The review suggests that new studies should focus on examining treatments specifically tailored for chemotherapy-induced diarrhea to address this gap in understanding and management. In individuals undergoing cyclophosphamide treatment, the detection of *Lactobacillus* spp., *Enterococcus hirae*, and *Barnesiella intestinihominis* (*B. intestinihominis*) was correlated with an enhanced treatment response [16]. The existing evidence endorsing the use of probiotics as supplementary therapy alongside anti-cancer treatments is constrained, particularly among cancer patients undergoing chemotherapy [17], especially in cases complicated by *P. aeruginosa* infection.

Pre-treatment with probiotics either as a single species or as combination of four species prolongs survival after experimental infection by multidrug-resistant *P. aeruginosa* in rodents partly by the prevention of bacterial translocation from the gut and attenuation of systemic inflammation [18]. Predominantly anaerobic resident microflora (e.g., *Bifidobacterium and Lactobacillus*) of the intestinal tract have a beneficial effect on colonization resistance and provide resistance toward invasion and/or outgrowth of potentially pathogenic bacteria (e.g., *Escherichia coli*, *enterobacteraceae*, *Pseudomonas*). Enteral administration of the *Bifidobacterium breve* strain Yakult could be an effective approach for achieving clinical benefits in immunocompromised hosts by improving their intestinal environments [10]. Matsumoto et al. showed that oral administration of *Bifidobacterium longum* could prevent the translocation of *Pseudomonas* from intestine in immunocompromised mice [19].

Hence, our study was structured to assess the impact of probiotics on the mucosal immune responses in the intestines of mice subjected to chemotherapy and that subsequently developed gut-derived *P. aeruginosa* sepsis.

## 2. Materials and Methods

### 2.1. Reagents

The cyclophosphamide was obtained from Sigma (St. Louis, MO, USA). Standard laboratory reagents were obtained from Sigma (St. Louis, MO, USA) or Fisher Scientific (Pittsburgh, PA, USA).

### 2.2. Bacterial Strains

The opportunistic pathogen, *P. aeruginosa* PAO1-LAC, was generously provided by Food Industry Research and Development Institute (FIRDI) in Taiwan. 

*P. aeruginosa* PAO1-LAC was grown for 2 h at 37 °C in Lysogeny broth supplemented with 50 ug/mL tetracycline, diluted 1:100 in fresh broth, and sub-cultured for 16 h at 37 °C under mild aeration. Then, the bacteria were washed twice in phosphate-buffered saline (PBS) and suspended in PBS to 10^8^ CFU/mL. *Lactobacillus rhamnosus* GG (LGG) (ATCC 53103) and *Bifidobacterium longum* (BL) (ATCC15707) were provided by the Food Industry Research and Development Institute (FIRDI, Taipei City 10015, Taiwan). LGG and BL were grown as per the manufacturer’s description. Solutions of LGG and BL were diluted 1:100 in fresh MRS broth and sub-cultured for 16 h at 37 °C under an anaerobic atmosphere, washed, and resuspend to a final concentration of 10^8^ CFU/mL.

### 2.3. Quantitative Real-Time PCR Analysis of Cecum RNA

Duplicate reactions were established, and a total of forty amplification cycles were conducted using an ABI 7500 Real-Time PCR System from Applied Biosystems (Waltham, MA, USA). Each cycle encompassed a denaturation step at 95 °C for 1 min, an annealing step at 54 °C for 1 min, and an extension step at 72 °C for 2 min. The threshold cycle (Ct), indicating the cycle at which the fluorescence surpasses a specific threshold value during the exponential amplification phase, was determined.

For analysis, the ABI7500 software (SDS V2.3) was utilized to acquire raw fluorescence data (Rn and DRn). The transcripts’ relative abundance was standardized against the quantity of β-actin transcript by subtracting the mean Ct value of β-actin from the mean Ct value of the target transcript for each experimental scenario. The disparity between the normalized Ct values of the infected cells and the control cells offers insight into the alteration in mRNA expression. Throughout the methodology and analysis, numerous elements of the MIQE guidelines were taken into account [20].

Upon collection, cecum samples were promptly frozen in liquid nitrogen and preserved at a temperature of −80 °C. To extract total RNA from the cecal tissue, TRI reagent (Ambio #15596018) and a Directzol RNA MiniPrep kit were utilized, following the guidelines provided by the manufacturer. Subsequently, the obtained RNA was subjected to reverse transcription to synthesize complementary DNA (cDNA). This cDNA was then employed in quantitative real-time PCR to assess and analyze the levels of mRNA expression. The primers for the mouse genes of interest and reaction protocol were set as in previous reports [16,18] except for mouse IL-17A, forward, 5′-ATCCCTCAAAGCTCAGCGTGTC-3′, reverse, 5′-GGGTCTTCATTGCGGTGGAGAG-3′; mouse IL-22, forward, 5′-GTCAACCGCACCTTTATGCT-3′, reverse, 5′-CATGTAGGGCTGGAACCTGT-3′; and mouse CRAMP, forward, 5′-GCCGCTGATTCTTTTGACAT-3′, reverse, 5′-GCCAAGGCAGGCCTACTACT-3′. The MIQE guidelines were taken into account for the methods and analysis [19].

### 2.4. Animal Experiments

Gut-derived *P. aeruginosa* sepsis in mice was induced as described previously [20]. All mice were generously provided by the National Laboratory Animal Center. Six-to-eight-week-old female specific-pathogen–free C57BL/6 mice were fed in an SPF room in the Kaohsiung Chang Gung Memorial Hospital animal center. Animal experiments were approved by the Kaohsiung Chang Gung Memorial Hospital Institutional Animal Care and Use Committee according to the legal requirements (see Figure S1).

During the sepsis model induction, mice were given the probiotics *Lactobacillus rhamnosus* GG (LGG) or *Bifidobacterium longum* (BL) via oral gavage and infected with 10^7^ CFU of *P, aeruginosa* PAO1-LAC (suspended in 100 μL PBS) or given 1xPBS buffer (100 μL sterile 1xPBS buffer for open control). Other groups were fed 100 μL of sterile 1xPBS (open control and PS group). Hence, the mice were divided into four groups: NA (open control), PS (comparison group), PS+LGG, and PS+BL. On the ninth and twelfth days, the mice were given intraperitoneal injections of 150–200 mg/kg of cyclophosphamide (infection group) or 1xPBS (open control). On the fourteenth day, the submandibular bleeding was collected by using lancets. Then, the mice were sacrificed by CO_2_ asphyxiation, and tissue samples from the spleens and livers were removed for the analysis of bacterial colonization and cytokines, chemokines, and AMP expressions, and samples of the cecum were snap-frozen in liquid nitrogen for the isolation of mRNA and fixed and embedded in paraffin for the assessment of disease activity and immunohistochemistry (IHC).

### 2.5. Histological Scores

Segments of the ileum, cecum, and colon were processed for fixation and paraffin embedding using established protocols. Alternatively, the tissue samples could be embedded in O.C.T. compound (Sakura, Torrance, CA, USA), rapidly frozen using liquid nitrogen, and subsequently stored at a temperature of −80 °C. The cryosections, either 5 or 30 μm in thickness, were placed onto glass slides, allowed to air dry for 2 h at room temperature, and then subjected to staining with hematoxylin and eosin (H&E) for visualization.

To ensure impartial evaluation, the tissue sections were anonymized before examination by a pathologist. The pathologist assigned scores to specific pathological alterations, including (i) neutrophil infiltration, (ii) mononuclear cell infiltration, (iii) submucosal edema, (iv) epithelial damage, and (v) presence of inflammatory exudate. These pathological changes were graded on a scale ranging from 0 to 4, denoting the following levels of severity: 0 for no changes, 1 for detectable changes, 2 for mild changes, 3 for moderate changes, and 4 for severe changes. For visual documentation, the images were captured using an Olympus BX41 microscope.

### 2.6. Analysis of P. aeruginosa Loads in Liver, Spleen, and Blood

The weight of all the tissue extracted from mice was measured and documented. Subsequently, the liver and spleen were gathered and placed in room-temperature PBS containing 1% Triton X-100 (Merck Ltd. Taipei, Taiwan). A micropestle was utilized to finely mince the tissue to the greatest extent possible. Blood was also collected for the *P. aeruginosa* culture. To estimate the number of *P. aeruginosa* colonized in a sample, serial dilutions were plated on LB agar plates with 50 μg/mL tetracycline. These plates were then cultured for 16 h at 37 °C under mild aeration.

### 2.7. Immunohistochemistry (IHC) Staining Analysis for Tight Junction Proteins

Thin sections of paraffin-embedded tissue samples extracted from the cecum of each animal were affixed onto glass slides. Subsequently, these slides underwent deparaffinization and rehydration before being subjected to microwave treatment in a retrieval buffer to facilitate antigen retrieval. Following this step, the slides were blocked using a solution composed of 10% normal serum and 1% bovine serum albumin in tris-buffered saline (TBS) for a duration of 2 h at room temperature. The slides were allowed to drain briefly before being subjected to overnight incubation at 4 °C with the primary antibody. Following this, the slides were rinsed multiple times with TBS buffer. Subsequently, they were incubated for 1 h at room temperature with a secondary antibody (HRP-conjugated antibody). Afterward, the slides underwent rinsing and were incubated with the chromogen (3,3′-diaminobenzidine) to visualize the target protein. Subsequently, they were counterstained with hematoxylin. Finally, a process of dehydration, clearing, and mounting was carried out to prepare the slides for further analysis.

A fully automated whole-slide scanning device (3DHISTECH, Sysmex, Hyogo, Japan) along with the associated software (Pannoramic viewer 1.15.4, Sysmex, Hyogo, Japan) was utilized for imaging purposes. The scanned images were subsequently subjected to analysis using a freely available software called ImageJ Fiji (Java 1.8.0_345). The semi-quantitative immunohistochemistry (IHC) technique (67) described in the bio-protocol by Wei Yue in 2019 is a robust method used for examining and assessing protein expression levels within tissue samples. Using the ImageJ Fiji software, we performed the deconvolution and subsequent analyses. A trained pathologist circled and measured the areas of interest within the scanned images. Ten regions of interest were selected from each slide image, and a minimum of three experiments were conducted to gather image values for subsequent statistical analysis.

### 2.8. Statistical Analysis

All the aforementioned experiments were conducted in triplicate, yielding consistent outcomes. Statistical analyses were carried out utilizing appropriate methods; the paired Student *t*-test or Mann–Whitney U test were used for comparing two parametric or nonparametric variables, and the Kruskal–Wallis one-way analysis of variance was used for comparing three or more nonparametric variables. The GraphPad Prism 8 software, developed by GraphPad Software in San Diego, CA, USA, was employed for these analyses. A significance threshold of *p* < 0.05 was adopted, indicating statistical significance.

## 3. Results

### 3.1. Probiotics Exerted Exaggeration of Bacterial Translocation in Chemotherapy-Induced Gut-Derived P. aeruginosa Sepsis in Mice

**Figure 1:**
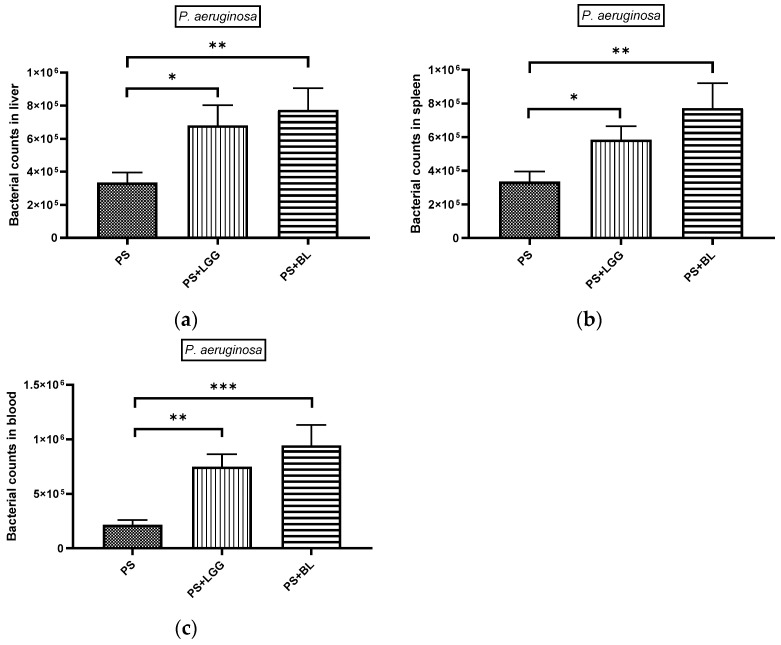
Probiotics exaggerate systemic translocation of gut-derived *P. aeruginosa* infection in mice receiving chemotherapy. After chemotherapy by cyclophosphamide, 6–8-week-old female C57BL/6 mice were incubated with 10^7^ CFU of *P. aeruginosa* (PAO1-LAC strain), along with vehicle, probiotics *Lactobacillus rhamnosus* GG (LGG), or *Bifidobacterium longum* (BL), daily, as shown in above protocol. Bacterial quantities were assessed in liver and spleen homogenates, as well as blood samples from mice undergoing chemotherapy and infected with bacteria. The presented data are expressed as the mean values with standard error of the mean (SEM) for bacterial load in both the liver (**a**), spleen (**b**), and blood (**c**). (*n* = 6). * *p* < 0.05, ** *p* < 0.01, *** *p* < 0.001.

To assess the effects of probiotics, specifically *Lactobacillus rhamnosus* GG (LGG) or *Bifidobacterium longum* (BL), on bacterial translocation to tissues and septic loads, we conducted experiments using mice with chemotherapy-induced gut-derived *P. aeruginosa* infections. In this study, we collected and homogenized liver and spleen samples and obtained blood samples. Subsequently, we plated these samples on LB plates to quantify the number of colony-forming units (CFU). Our findings revealed that the administration of probiotics, either the LGG or BL regimen, led to an increase in the bacterial loads in the liver, spleen, and bloodstream of the mice with gut-derived *P. aeruginosa* infections undergoing chemotherapy (Figure 1).

### 3.2. Probiotics Exaggerated the Severity of Colitis in Mice Undergoing Chemotherapy and Suffering from Gut-Derived P. aeruginosa Sepsis

**Figure 2:**
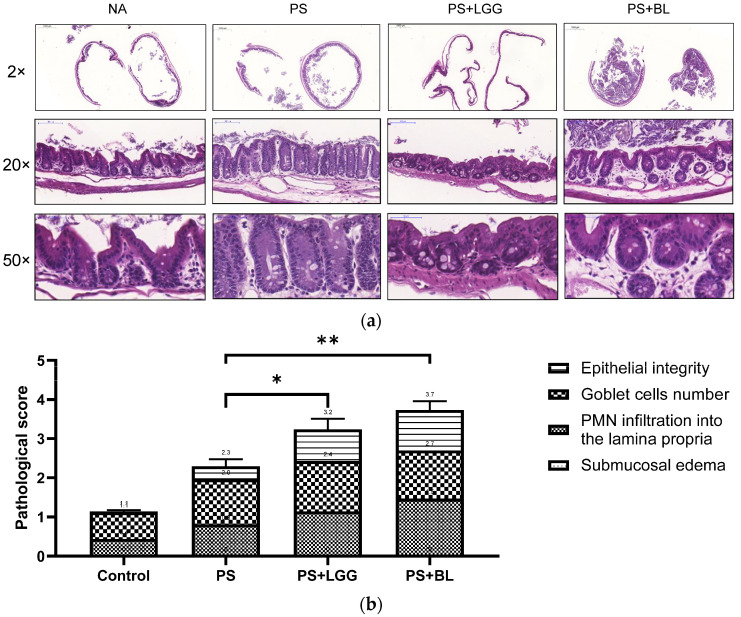
Probiotics exaggerated the severity of *P. aeruginosa*-infected colitis in mice receiving chemotherapy. Six-to-eight-week-old female C57BL/6 mice (Charles River, MA, USA) were bred and housed under specific-pathogen-free conditions in the animal facility of the Center for Cellular and Biomolecular Research, Kaohsiung, Taiwan. After chemotherapy by cyclophosphamide, mice were incubated with 10^7^ CFU of *P. aeruginosa* (PAO1-LAC strain), along with vehicle, probiotics *Lactobacillus rhamnosus* GG (LGG), or *Bifidobacterium longum* (BL)*,* daily, as shown in above protocol. The cecum was surgically removed and subsequently preserved in formaldehyde. Following this, tissue sections were stained using the hematoxylin and eosin (H&E) staining technique. We present representative histological images captured at 2×, 20× and 50× magnification (**a**), depicting the cecum samples obtained from the experimental mice. Subsequently, we quantified the histological scores to assess the severity of colitis (**b**) based on the analysis of the H&E-stained images. The data shown are means ± SEM (*n* = 6 mice/group). * *p* < 0.05, ** *p* < 0.01.

To investigate the impact of probiotics on the severity of *P. aeruginosa*-induced colitis in mice undergoing chemotherapy, we examined the cecal pathology of wild-type (WT) mice that had been infected with *P. aeruginosa* and treated with cyclophosphamide, both with and without the administration of the probiotics LGG or the BL regimen. Our assessment of cecal sections stained with H&E revealed noticeable pathological alterations in the infected WT mice, consistent with a previous study [20], as depicted in Figure 2a. Surprisingly, our analysis indicated that the probiotics LGG or BL exacerbated the severity of *P. aeruginosa*-induced colitis. When utilizing histological scoring, we observed that the administration of the probiotics LGG or BL led to significantly higher colitis severity scores compared to those of the WT mice infected with *P. aeruginosa* but not treated with the probiotics (Figure 2b).

### 3.3. Probiotics LGG and BL Exaggerated Cecal Inflammatory Responses but Decreased Antimicrobial Peptide in Mice Undergoing Chemotherapy and Suffering from Gut-Derived P. aeruginosa Sepsis

**Figure 3:**
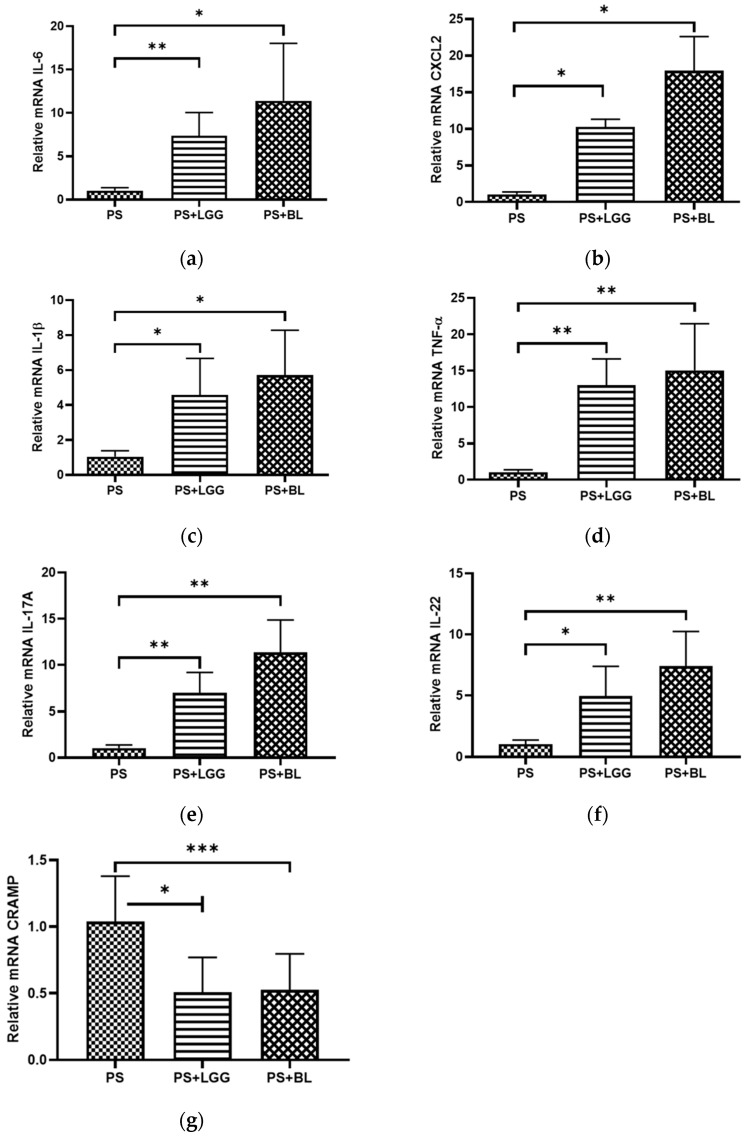
The immunoregulatory effects of probiotics on cecal cytokines and antimicrobial peptide in mice undergoing chemotherapy and suffering from gut-derived *P. aeruginosa* sepsis. Following *P. aeruginosa* infection, we collected cecal tissues from various groups, including the control (NA), *P. aeruginosa*-infected (PS), and mice infected with *P. aeruginosa* and treated with probiotics *Lactobacillus rhamnosus* GG (LGG) or *Bifidobacterium longum* (BL). From these cecal tissues, we extracted total RNA. Subsequently, we conducted real-time quantitative PCR to analyze the mRNA expressions of IL-6 (**a**), CXCL2 (**b**), IL-1β (**c**), TNF-α (**d**), IL-17a (**e**), IL-22 (**f**), and CRAMP (**g**). The values obtained were measured as fold increases when compared to the mRNA levels observed in mice infected solely with Salmonella, serving as the reference group. The data shown are means ± the SEM (*n* = 6 mice/group). An asterisk indicates significant differences among groups based on one-way ANOVA. * p < 0.05, ** p < 0.01, *** *p* < 0.001.

To explore the impact of the probiotics LGG or BL on the inflammatory and antimicrobial peptide responses in mice with *P. aeruginosa*-infected colitis following chemotherapy, we assessed the gene expression levels of cytokines and antimicrobial peptides using real-time PCR in the cecal tissue. Our results demonstrated that the cecal gene expression of IL-6, CXCL2 (murine IL-8 homologue), IL-1β, TNF-α, IL-17a, IL-22, and CRAMP (homologue of human cathelicidin LL-37) (as shown in Figure 3) was significantly elevated in the mice with *P. aeruginosa*-infected colitis after chemotherapy. Interestingly, when we treated the infected colitis mice with LGG or BL, we observed a significant increase in IL-6, CXCL2, IL-1β, TNF-α, IL-17a, and IL-22 gene expression in the cecal tissue, indicating an enhanced inflammatory response. However, the gene expression of CRAMP was significantly reduced under these probiotic treatment conditions.

Altogether, these results suggest the detrimental effects of LGG or BL on the translocation of bacterial loads and severity of *P. aeruginosa*-infected colitis in chemotherapy-treated mice by decreasing anti-bacterial and anti-inflammatory responses.

### 3.4. Admixture of Probiotics Enhanced the Tight Junction Protein Expression in Cecal Mucosa of Mice Undergoing Chemotherapy and Suffering from Gut-Derived P. aeruginosa Sepsis 

Recently, we observed that *Salmonella* infection induced significantly enhanced zonulin and claudin-2, resulting in an increased bacterial invasion and translocation [21]. Hence, we conducted an investigation into the expression of zonulin and claudin-2 proteins within the cecal mucosa of a gut-derived *P. aeruginosa* sepsis mouse model treated with LGG or BL. We employed immunohistochemistry staining to analyze the protein expression of zonulin and claudin-2 in the cecal tissue of mice infected with *P. aeruginosa* following chemotherapy, both with and without the administration of the LGG or BL regimen.

**Figure 4:**
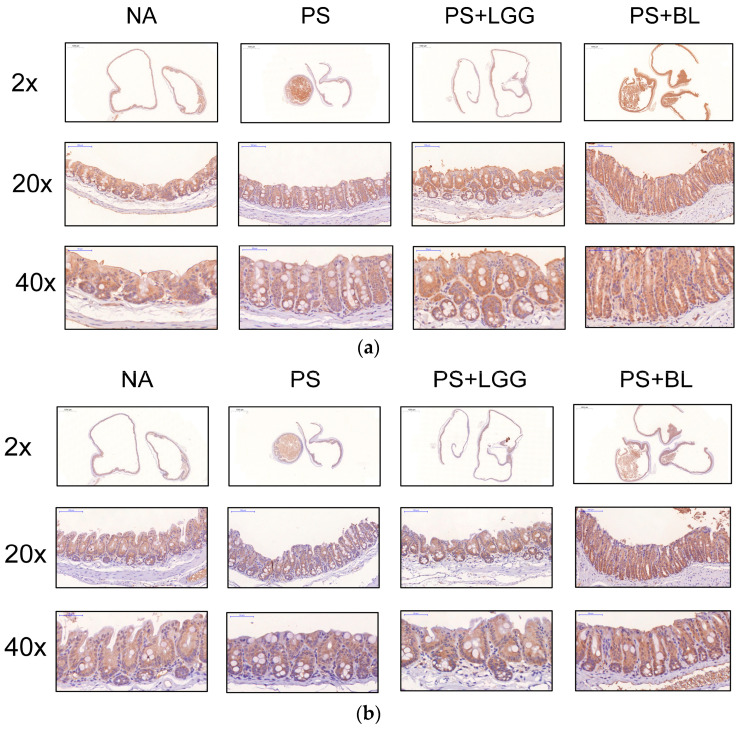

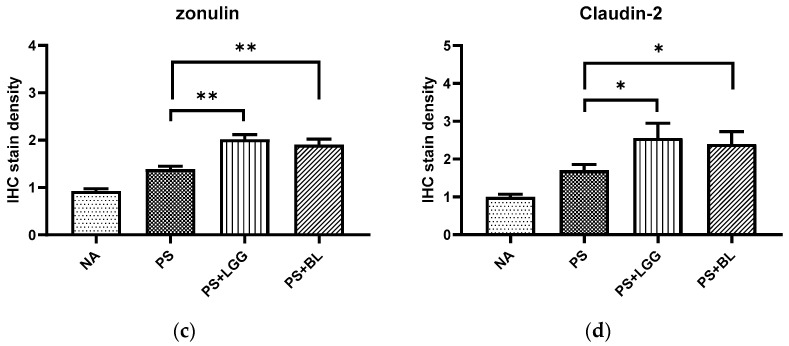
Probiotics increased the colon epithelial zonulin and claudin-2 protein expression in mice undergoing chemotherapy and suffering from gut-derived *P. aeruginosa* sepsis. Mice were treated or infected as described in the material and methods. After *P. aeruginosa* infection, cecal tissues were obtained from control (NA), *P. aeruginosa* -infected (PS), and probiotics *Lactobacillus rhamnosus* GG (LGG)- or *Bifidobacterium longum* (BL)-treated infected mice (LGG+PS, BL+PS). Zonulin (**a**) and claudin-2 (**b**) protein expression in these groups were detected by immunohistochemistry staining at 2×, 20× and 40× magnification (*n* = 3). The levels of zonulin (**c**) and claudin-2 (**d**) immunohistochemistry staining were analyzed and calculated by imageJ (Java 1.8.0_345). * p < 0.05, ** p< 0.01.

As shown in Figure 4, our results revealed that zonulin and claudin-2 were present at significantly increased levels in the cecal mucosa in the *P. aeruginosa*-infected colitis mice via the treatment of LGG or BL compared with the sham group. This suggests that LGG or BL exaggerated the bacterial translocation by increasing the mucosal zonulin and claudin-2 protein expression in the cecum of the mice undergoing chemotherapy and suffering from gut-derived *P. aeruginosa* sepsis.

## 4. Discussion

In a study conducted on a murine model, it was observed that the administration of probiotics resulted in sepsis. This led to an increase in the adherence of the intestinal mucosa, consequently amplifying the virulence and the translocation of bacteria. Consequently, the pathogenicity of potent probiotics might be heightened [22]. Another report examined athymic adult mice treated with various probiotic bacteria and found that neonatal mice treated with *L. reuteri* probiotics experienced an increased mortality rate [23]. This study also hypothesized that the underdeveloped immune system in neonatal mice might contribute to a heightened risk of probiotic-induced sepsis. Numerous factors contribute to the development of probiotic-induced sepsis, including chronic illnesses, compromised immune function, changes in the integrity of the epithelial barrier, and the amplification of bacterial resistance. Lactic acid bacteria and *bifidobacteria* carrying antibiotic resistance genes have the potential to transfer these genes to pathogenic and opportunistic bacteria found in humans, such as *Staphylococcus aureus* and *enterococci* [24]. The utilization of probiotics could disrupt the equilibrium of the microbiota, potentially affecting the nutritional levels within the subject. Such alterations in nutrition might contribute to the development of sepsis [25]. The reconstitution of the gut mucosal microbiome after antibiotic treatment is hindered by the use of probiotics [26]. Imbalance or dysregulation of the gut microbial flora might exacerbate sepsis by triggering the production of proinflammatory mediators [27]. The onset of sepsis involves the disruption of intestinal barrier function, changes in the gut microbiota, and the translocation of the intestinal microbiome, culminating in systemic and localized inflammatory responses. These responses subsequently modify immune homeostasis within the systemic environment [27]. 

The administration of cyclophosphamide diminishes tight junctions and adherens junctions, thereby causing impairment to the mucosal barrier. This leads to heightened intestinal permeability and facilitates the invasion of potentially pathogenic bacteria [12]. Consequently, reinforcing the mechanical mucosal barrier and bolstering colonization resistance could present promising strategies for application in cancer patients, aiming to mitigate infectious complications. Pathogenic infections, compromised gut motility, and various factors such as diet, medications, and stress can contribute to the development of a condition known as “leaky gut,” which is necessary for widespread gut inflammation to manifest. Probiotics have demonstrated a significant capacity to enhance gut barrier function and prevent gut permeability. However, in situations where the gut is already permeable, as seen in animal models of colitis induced by the chemical dextran sodium sulfate (DSS), probiotics may potentially provoke gut inflammation [28]. Indeed, probiotics possess the ability to traverse a disrupted gut barrier, reaching the submucosal compartment where they can stimulate the release of pro-inflammatory cytokines. When the gut is relatively healthy, probiotics are capable of reinforcing the gut barrier and shielding it against potential damage. However, in cases where the gut barrier is compromised, certain probiotic strains might exacerbate inflammation. For example, research has demonstrated that strains like *Lactobacillus rhamnosus* GG and *Lactobacillus plantarum* NCIMB8826 have the potential to worsen gut inflammation.

Certainly, the effects of probiotics in clinical applications vary significantly. For instance, one specific combination of a probiotic with a prebiotic (symbiotic) was discovered to effectively prevent sepsis in infants [29], while another formulation failed to prevent necrotizing enterocolitis in very preterm infants [30]. Individuals in neonatal stages and/or those with certain clinical conditions such as malignancies, leaky gut, diabetes mellitus, and during post-organ transplant recovery might not experience the anticipated benefits from probiotics [31]. In certain vulnerable groups with weakened immunity, certain probiotic strains have the potential to exploit this weakened state and transform into opportunistic pathogens. This can lead to clinical infections, including severe conditions such as life-threatening pneumonia, endocarditis, and sepsis [32]. Additionally, the uncontrolled and widespread utilization of probiotics bears the potential risk of transferring antibiotic resistance through plasmids to infectious pathogens in the gut [33]. From 1995 to June 2021, a total of 49 case reports of probiotic-related infections were identified. These infections were attributed to various strains, with *Lactobacillus* spp. accounting for 35%, *Saccharomyces* spp. for 29%, *Bifidobacterium* spp. for 31%, *Bacillus clausii* for 4%, and *Escherichia coli* for 2% [34]. A significant majority of patients (80%) affected were younger than 2 years old, and sepsis was the most commonly observed condition (69.4%) [34]. In this context, prematurity (55%) and the use of intravenous catheters (51%) were identified as the most prevalent predisposing factors. Tragically, three children (6%) succumbed to these infections. Within the lactobacilli strains, *L. rhamnosus* strains were notably linked to human sepsis, indicating a heightened potential for translocation [35]. Additionally, certain individuals with leaky gut, characterized by compromised mucosal integrity, are more susceptible to invasion, thereby increasing the risk of infections and potentially sepsis [36].

Gut injury possesses the potential not only to amplify local damage but also to induce distant injury and organ failure. Critical illnesses trigger intestinal hyperpermeability, allowing intact bacteria to translocate into the systemic circulation, culminating in sepsis and subsequent organ failure. The regulation of permeability is governed by tight junction proteins that manage the paracellular space between each cell [37]. Furthermore, various enteric pathogens, including commensal *Escherichia coli*, laboratory *E. coli*, virulent *E. coli*, and *Salmonella typhi*, have demonstrated the ability to trigger the release of zonulin from the intestine [38]. Li et al. discovered that the exposure of Caco-2 cells to *Pseudomonas fluorescens* resulted in an increased expression of zonulin [39]. This inappropriately heightened production of zonulin, which is considered a potential regulator of tight junction permeability, leads to a functional breakdown of barrier function. Consequently, this breakdown allows for an uncontrolled influx of microbial antigens, initiating an innate immune response within the submucosal immune compartment [40]. Continuation of this process triggers the initiation of an adaptive immune response, prompting the production of pro-inflammatory cytokines such as IFN-γ and TNF-α. These cytokines further widen the paracellular pathway, allowing for the passage of antigens, thus perpetuating a detrimental cycle. In conditions like the DSS colitis model, zonulin-dependent impairment of the intestinal barrier represents an initial step leading to altered gut permeability. This alteration is associated with heightened morbidity and mortality [41].

Intestinal barrier dysfunction has been identified as a significant factor in the development and advancement of septicemia. Yoseph et al. conducted research demonstrating an altered expression of tight junction proteins in an experimental model of sepsis [42]. Additionally, in patients diagnosed with septicemia, elevated serum levels of zonulin were observed [43]. Dysbiosis of the microbiome has the potential to initiate the release of zonulin, thereby allowing contents from the intestinal lumen to breach the epithelial barrier. Consequently, this breach results in the release of proinflammatory cytokines. In the study by Liu et al. [44], it was shown that the risk of sepsis correlates with a reduction in serum zonulin levels. Elevated zonulin levels are indicative of damage to the barrier and a loss of regulation over the passage of pathogenic microorganisms from the intestinal lumen into the bloodstream [45]. Additionally, the research suggests that heightened levels of zonulin released from enterocytes facilitate the migration of bacteria across the epithelium, potentially contributing to the progression of sepsis. Numerous studies have highlighted the notable impact of probiotics in reducing serum zonulin levels [46], but the findings remain inconclusive. The induction of IL-22, a factor demonstrated to bolster host defense against microbes [47] and fortify epithelial barrier integrity [48], played a pivotal role in regulating microbial dysbiosis associated with colitis and sepsis. Conversely, IL-22 could also potentially contribute to inflammatory damage in the epithelium by stimulating the release of chemokines that recruit polymorphonuclear leukocytes (PMNs) [49]. Indeed, Sonnenberg et al. previously demonstrated that during bleomycin-induced acute lung injury, the tissue-protective effects of IL-22 are overshadowed by its pro-inflammatory characteristics, attributed to its synergistic actions with IL-17, leading to the recruitment of PMNs [49,50]. This supports the notion of the dual functionality of IL-22 during inflammation and emphasizes the critical balance between the protective and detrimental effects of IL-22 on epithelial cells. Sepsis is marked by an initial and intense systemic inflammatory response syndrome (SIRS), commonly referred to as a cytokine storm, characterized by overexpression of inflammatory mediators like IL-1β, IL-6, and IL-8. Elevated levels of these cytokines contribute to tissue damage and progressive organ dysfunction. In our study, we observed that probiotics led to increased zonulin staining on the colon mucosa of mice with gut-derived *P. aeruginosa* sepsis. This increase in zonulin was associated with heightened colitis scores, escalated proinflammatory responses, decreased antimicrobial peptide LL-37, and increased invasiveness of the microorganism.

## 5. Conclusions

*P. aeruginosa* presents a high fatality rate among Gram-negative bacteria and is a prevalent hospital-acquired infection. Moreover, the emergence of multi-drug-resistant strains exacerbates its threat. Using a chemotherapy-induced gut-derived *P. aeruginosa* sepsis animal model, our study highlighted the risks associated with probiotics in mice receiving chemotherapy. This research underscores the potential hazards of probiotic administration when the intestinal barrier function is significantly compromised, especially in cases of chemotherapy-induced gut-derived *P. aeruginosa* sepsis. These findings suggest the need for caution in employing probiotics under such conditions. Furthermore, this understanding can be extended to investigate various treatment strategies in the context of chemotherapy-induced gut-derived *P. aeruginosa* sepsis.

## Data Availability

The data presented in this study are available on request from the corresponding author.

## Supplementary Materials

The following supporting information can be downloaded at: https://www.mdpi.com/article/10.3390/biomedicines12020253/s1, Figure S1: Experimental groups and protocol.
