# Supplementary material for: The Hazards of Probiotics on Gut-Derived Pseudomonas aeruginosa Sepsis in Mice Undergoing Chemotherapy
Source: Biomedicines. 2024 Jan 23;12(2):253. doi: 10.3390/biomedicines12020253 (PMC10886725; doi:10.3390/biomedicines12020253)
Supplement: Supplementary file 1 [file biomedicines-12-00253-s001.zip › biomedicines-2763027-supplementary.pptx]

## Slide 1
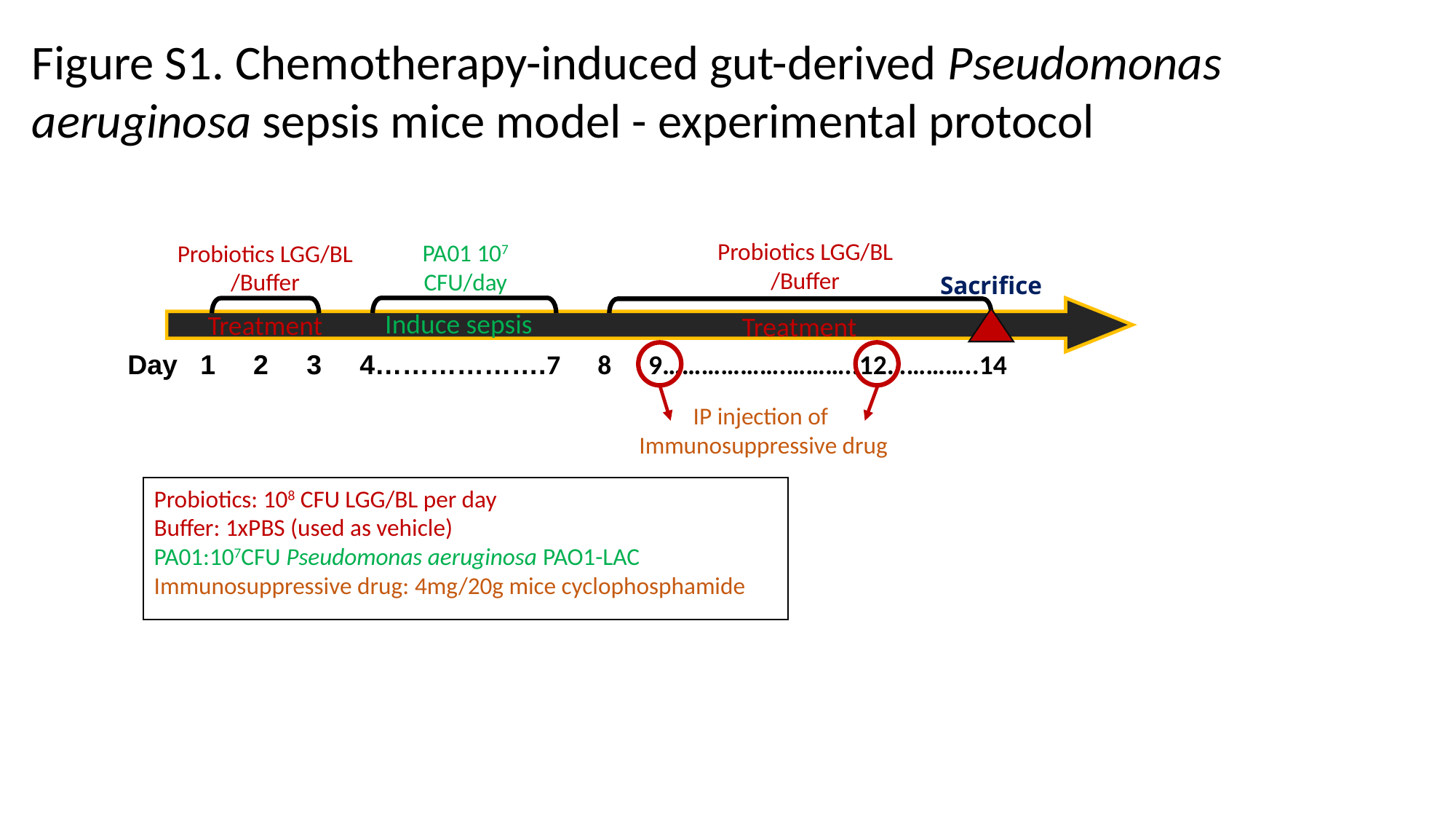

Figure S1. Chemotherapy-induced gut-derived Pseudomonas aeruginosa sepsis mice model - experimental protocol
Probiotics LGG/BL
/Buffer
PA01 107 CFU/day
Sacrifice
Induce sepsis
Treatment
Treatment
IP injection of
Immunosuppressive drug
Probiotics: 108 CFU LGG/BL per day
Buffer: 1xPBS (used as vehicle)
PA01:107CFU Pseudomonas aeruginosa PAO1-LAC
Immunosuppressive drug: 4mg/20g mice cyclophosphamide
Probiotics LGG/BL
/Buffer
Day 1 2 3 4……………….7 8 9……………….………..12…………..14
